# Are there any concerns about the usage of biological agents in psoriasis?

**DOI:** 10.55730/1300-0144.5860

**Published:** 2024-06-01

**Authors:** Burhan ENGİN, Eylül KASAPOĞLU

**Affiliations:** Department of Dermatology, Faculty of Medicine, İstanbul University-Cerrahpaşa, İstanbul, Turkiye

**To the Editor**,

Psoriasis is a common chronic skin disorder that can have significant effects on quality of life. Numerous topical and systemic therapies are available for the treatment of cutaneous manifestations of psoriasis. Treatment modalities are chosen on the basis of disease severity, relevant comorbidities, patient preference, efficacy, and evaluation of individual patient response. Biological agents are important treatment options for moderate-to-severe plaque-type psoriasis. The available biological agents for psoriasis have excellent short-term and long-term efficacy and favorable tolerability. However, in our clinical practice, we observed that some of our patients initially feared biological agent therapy. The phobia of biological agents is generally promoted by misinformation and involves erroneous beliefs and vague negative feelings about biological agents. We evaluated 405 psoriasis patients registered with the Cerrahpaşa Faculty of Medicine Psoriasis Follow-up and Treatment Clinic who used biological agents. Among these patients, eight were initially afraid of using biological agents. Despite the high initial Psoriasis Area and Severity Index and Dermatology Life Quality Index scores of these patients, they hesitated to undergo biological agent therapy. The demographic data of these patients are presented in Table 1 and their reasons for biological agent phobia are detailed in Table 2. These tables contain the data of patients who started and were followed up with biological agent treatment. In addition, two patients who initially declined biological agent treatment due to concerns regarding the terminology of “biological agents” were followed with methotrexate treatment. After initiating biological agent treatment, none of the patients discontinued it due to further phobia.

All eight patients indicated a need for reassurance regarding biological agents and expressed trepidation specifically about the term “biological agents.” Anti-tumor necrosis factor alpha (TNF-α) agents were planned as the initial biological treatment for six of these patients. These were their first biological agents. Their fear likely stemmed from a lack of knowledge about biological agents at the time. Furthermore, all patients expressed a desire to discontinue treatment as soon as possible, showing hesitation upon learning that biological agent therapy may necessitate long-term use.

Some patients were reluctant to undergo biological agent therapy due to concerns about infection risks. They feared the potential impact on their immune systems and preferred medications that would not compromise their immunity. It is noteworthy that none of these patients were treated during the COVID-19 era; they began treatment either before or after the pandemic. Consequently, it appears that common infections such as influenza and acute upper respiratory and urinary tract infections are significant concerns for patients at the initiation of biological treatment. We tell all of our patients about the risk of infection before starting treatment.

Additionally, some patients feared that biological agents would induce weight gain, a concern shared by individuals with both high and normal body mass index values. Most patients reported biological agent phobia in relation to anti-TNF-α treatment, possibly influenced by information suggesting potential weight gain associated with such agents. Another contributing factor may have been the conflation of biological agents with systemic corticosteroids, a common misconception in Türkiye, where patients often misunderstand systemic treatments to include steroids.

A subset of patients expressed concerns about potential organ damage from biological agent therapy. This apprehension may have arisen from the extensive screening tests required for liver and pulmonary function, as well as infection screenings (e.g., for tuberculosis and hepatitis) prior to treatment initiation. However, these patients were convinced after we provided the necessary explanations.

We aim to share our observations regarding the phobia of biological agents and its possible causes. Similar to the existing literature on corticophobia among parents of children with atopic dermatitis, wherein a self-administered questionnaire was developed to yield Topical Corticosteroid Phobia (TOPICOP) scores and thus measure corticophobia, a comparable questionnaire could be devised to assess phobias of biological agents [1].

## Figures and Tables

**Table 1:** Demographic data of patients.

Patient number	Age	Sex	Previous treatments	Initial PASI	Initial DLQI	Biological agent
1	35	M	Topical agents, MTX	30	24	Adalimumab
2	39	F	PUVA, MTX, cyclosporine	33	26	Ustekinumab
3	46	M	PUVA, MTX, cyclosporine	47	29	Adalimumab
4	33	M	PUVA, MTX	24	20	Adalimumab
5	39	F	PUVA, MTX	15	22	Adalimumab
6	45	M	MTX, cyclosporine	27	23	Adalimumab
7	29	M	MTX	39	29	Infliximab
8	25	M	PUVA, MTX	41	23	Ustekinumab

MTX: Methotrexate; PASI: Psoriasis Area and Severity Index; DLQI: Dermatology Life Quality Index

**Table 2:** Patients’ answers regarding the reasons for phobia of biological agents.

Reason	Patients’ numbers
I need reassurance about biological agents.	1, 2, 3, 4, 5, 6, 7, 8
I am afraid of the term “biological agent.”	1, 2, 3, 4, 5, 6, 7, 8
I want to stop the treatment as soon as I can.	1, 2, 3, 4, 5, 6, 7, 8
Biological agents can lead to infections.	1, 2, 4
Biological agents will make me fat.	3, 4, 7, 8
Biologic agents will damage my organs.	3, 5, 6, 7

## Data Availability

Data are available on request from the authors.
